# A personalized federated hypernetworks based aggregation approach for intrusion detection systems

**DOI:** 10.1038/s41598-025-11659-7

**Published:** 2025-09-30

**Authors:** Chunduru Sri Abhijit, Y. Annie Jerusha, S. P. Syed Ibrahim, Vijay Varadharajan

**Affiliations:** 1 School of Computer Science and Engineering, Vellore Institute of Technology, Chennai Campus, Chennai, 600127 India; 2 Advanced Cyber Security Engineering Research Centre, The University of Newcastle, Callaghan, Australia

**Keywords:** Federated learning (FL), Network intrusion detection system (NIDS), Non-independent and identically distributed (Non-IID) data, Innovation, Internet of Things (IoT), Hypernetworks, Computational science, Computer science

## Abstract

Traditional network intrusion detection systems (NIDS) face significant scalability challenges due to the vast amount of data generated by Internet of Things (IoT) devices, compounded by growing privacy concerns. Federated Learning (FL) has emerged as a promising solution, offering a distributed, privacy-preserving paradigm that enables Deep Learning (DL) models to be trained locally, thereby mitigating privacy risks associated with centralized data processing. However, conventional FL strategies come with inherent limitations. First, they require all clients to use the same model architecture, making personalized learning difficult particularly in Non-Independent and Identically Distributed (Non-IID) heterogeneous data settings. Second, the weight aggregation process in FL introduces significant communication overhead, potentially slowing down training. While encryption techniques such as homomorphic encryption and differential privacy enhance security, they also increase computational costs and can still reveal data distribution patterns if compromised. These challenges are further exacerbated in dynamic IoT environments, where evolving attack types continuously alter data distributions. To address these issues, we propose a Personalized Federated Hypernetworks-based aggregation strategy for Intrusion Detection Systems (PerFedHypID). Unlike conventional FL approaches that rely on weight-based aggregation, PerFedHypID utilizes embedding vectors, which are computationally lighter and enable enhanced personalization. Our method leverages personalized layers and hypernetwork-based aggregation to achieve both efficiency and adaptability. We extensively evaluate PerFedHypID on the CSE-CICIDS-2018 and UNSW-NB-15 datasets under various non-IID heterogeneous settings. The results demonstrate that our approach outperforms state-of-the-art personalized federated learning algorithms, offering robust performance and improved adaptability in dynamic IoT environments.

## Introduction

The landscape of Machine Learning (ML) is experiencing a paradigm shift with the advent of Federated Learning (FL), an approach that addresses critical challenges related to privacy, scalability, and collaboration in distributed data environments. Unlike traditional ML methods that require centralizing sensitive data, FL enables collaborative model training while ensuring data remains decentralized. This makes FL particularly suitable for applications in healthcare, finance, smart grids, and natural language processing^1^. The increasing significance of FL is reflected in numerous surveys and research studies that highlight its growing adoption across diverse domains^2–4^. Real-world implementations, such as healthcare data analysis and federated reinforcement learning, showcase FL’s ability to achieve high-performance results while preserving data privacy^5,6^.

Despite its advantages, conventional FL approaches face key limitations, particularly in terms of personalization. Traditional FL frameworks often rely on global aggregation of model weights, which, while facilitating collaborative learning, can lead to suboptimal performance for individual clients. This issue is especially pronounced in Intrusion Detection Systems (IDS), where network traffic behavior is dynamic and constantly evolving. FL models trained on aggregated data struggle to adapt to these changes, leading to a decline in detection accuracy over time^7^. Additionally, weight-based aggregation methods introduce substantial computational overhead, reducing the scalability and efficiency of FL, particularly when handling large and complex datasets from modern network environments^8^. Existing FL techniques predominantly focus on aggregating weight updates while overlooking the critical need for personalized model training. However, effective intrusion detection requires customization to account for unique data characteristics across different clients, as even subtle variations in network traffic may signal potential security threats^9^.

Traditional centralized Network Intrusion Detection Systems (NIDS) further exacerbate these challenges. These systems rely on a central server for data processing and model building, necessitating the transmission of sensitive network data. This centralized approach poses serious privacy and security concerns, as sharing sensitive network data increases the risk of exposure and breaches. Furthermore, transmitting large volumes of network traffic data is computationally expensive and can lead to system bottlenecks, limiting overall performance.

One commonly used technique in collaborative learning is the exchange of gradients, which was traditionally believed to be safe for sharing, as it was assumed that training data could not be reconstructed from gradients. However, research has demonstrated that it is indeed possible to reconstruct confidential training data from shared gradients^10^. This finding underscores the urgent need for more secure and efficient approaches in federated intrusion detection.

To address these challenges, we introduce PerFedHypID, a novel FL-based intrusion detection approach that enhances personalization and efficiency. PerFedHypID leverages Hypernetworks^11^ to transmit a minimal set of parameters for personalized training, optimizing communication and computational overhead. Hypernetworks enable the efficient generation of parameters for another network, improving the adaptability of FL models to diverse network environments.

In addition, PerFedHypID employs an embedding-based representation approach to handle data heterogeneity in FL. By using embedding vectors to capture diverse characteristics of different network traffic patterns, clients can share relevant information while maintaining privacy. This approach allows FL participants to learn from each other’s strengths, improving detection accuracy. Rather than transmitting full model parameters, PerFedHypID utilizes a hypernetwork to generate model updates based on learnable embedding vectors, enhancing both efficiency and security. By structuring the embedding vector space as a low-dimensional manifold, the hypernetwork effectively generalizes across different client models, optimizing performance while minimizing communication overhead^3^.

PerFedHypID addresses the limitations of traditional FL in intrusion detection through the following key innovations:

1. Personalized Model Parameters: PerFedHypID generates personalized model parameters for each client, allowing for better adaptation to specific network environments and data characteristics.
2. Hypernetwork-Based Adaptation: By employing hypernetworks, PerFedHypID enhances local model components, enabling the system to learn complex relationships within the data for improved intrusion detection.
3. Embedding-Based Aggregation: PerFedHypID utilizes embeddings-based aggregation methods for efficient and privacy-preserving communication between clients and the central server.

By focusing on personalization and efficient communication, PerFedHypID presents a robust solution for intrusion detection in an evolving cyber threat landscape. The subsequent sections will delve deeper into the limitations of traditional FL for IDS, provide a comprehensive overview of the PerFedHypID architecture, and evaluate its effectiveness in comparison to existing approaches. Through an emphasis on personalized learning and efficient communication, PerFedHypID offers a scalable and adaptable solution to counter the dynamic nature of cyber threats.

## Related work

Federated Learning (FL) has been extensively explored in the context of Network Intrusion Detection Systems (NIDS) to enhance network security while preserving data privacy. This section categorizes the related work into four key themes:

1. FL-based NIDS frameworks.
2. Privacy-enhancing and communication-efficient FL.
3. Handling data heterogeneity and class imbalance in FL, and
4. Personalized Federated Learning (PFL) approaches for intrusion detection.

### FL-based NIDS frameworks

Several studies have proposed FL frameworks for intrusion detection, integrating machine learning models with distributed training methodologies. Tian Dong et al.^12^ introduced FEDFOREST, a federated learning-based NIDS that integrates Gradient Boosting Decision Trees (GBDT) for improved interpretability and efficiency. Their approach enables local clients to extract cyberattack features while preserving privacy, demonstrating superior performance across multiple cyberattack datasets. Similarly, Md Mamunur Rashid et al.^13^ proposed a Federated Learning (FL)-based intrusion detection system for IoT networks, preserving data privacy by training models locally on IoT devices. Their approach, evaluated on the Edge-IIoTset dataset, achieves 92.49% accuracy, closely matching centralized ML models while enhancing security and scalability. These studies highlight the effectiveness of FL in real-world IoT settings while demonstrating comparable accuracy to centralized approaches.

Jianbin Li et al.^14^ proposed DAFL, which enhances NIDS by integrating FL with dynamic filtering and weighting mechanisms. DAFL mitigates unreliable attack data and reduces transmission overhead by 33–71%. Likewise, Pratyay Kumar et al.^15^ introduced FLNET2023, a federated learning framework trained on the CORE emulation benchmark dataset, emphasizing local class imbalance concerns. These frameworks demonstrate the need for adaptive and efficient FL solutions for NIDS to handle real-world traffic.

#### Privacy-enhancing and communication-efficient FL

Addressing privacy concerns and reducing communication overhead are crucial aspects of FL-based NIDS. Abhijit et al.^16^ analyzed multiple ML algorithms on the UNSW-NB15 dataset and emphasized adopting FL to overcome the limitations of centralized methods. Meryem Janati Idrissi et al.^17^ proposed Fed-ANIDS, an autoencoder-based IDS within an FL framework (FedProx). Compared to GAN-based models, Fed-ANIDS enhances detection while reducing false alarms. These studies reinforce the significance of privacy-preserving FL models while maintaining performance.

Weixiang Han et al.^18^ introduced Clustering-enabled Federated MetaTraining (CFMT) for AIoT intrusion detection. CFMT mitigates class imbalance and statistical heterogeneity by leveraging federated clustering. Efforts like CFMT highlight the importance of reducing FL communication costs while improving model robustness.

#### Handling data heterogeneity and class imbalance in FL

One of the biggest challenges in FL-based NIDS is the presence of heterogeneous and imbalanced data across clients. Vasiliki Kelli et al.^19^ incorporated active learning into FL to improve model accuracy with fewer labeled samples. The study demonstrated that even 10–20 active learning queries significantly enhance local model performance across different dataset biases. Jihao Yang et al.^20^ developed hierarchical federated learning using hypernetworks (HN) to improve accuracy, communication efficiency, and computational cost. By addressing statistical heterogeneity, these methods improve FL generalization across diverse client data distributions.

Su et al.^21^ introduced APFed, a collaborative IDS for Maritime Meteorological Sensor Networks, balancing localization and generalization using Lightweight Group Convolutional Neural Networks (LGCNN). Evaluations on NSL-KDD demonstrate APFed’s ability to detect both known and unknown attacks. By improving robustness in non-IID settings, these techniques ensure better adaptability of FL models.

#### Personalized federated learning (PFL) for intrusion detection

Personalized FL (PFL) offers client-specific model updates to enhance adaptability and mitigate data heterogeneity challenges. Rakotomamonjy et al.^22^ categorized PFL into global model optimization and client-side customization, employing methods like pFedMe^23^, Ditto^24^, and FedProx^25^. PerFed^26^ and FedBabu^27^ further enhance PFL by partitioning models into shared and client-specific components.

Huang et al.^28^ introduced EEFED, an FL framework that enables personalized updates to improve attack detection. EEFED utilizes an optimal backtracking parameter strategy to handle non-IID data. The first application of hypernetworks in FL, pFedHN^29^, allows parameter sharing while preserving personalized user models.

We propose PerFedHypID, a novel hypernetwork-based aggregation technique for intrusion detection using embedding vectors. Unlike conventional FL methods that aggregate global weights, PerFedHypID dynamically generates individualized model weights for each client, enhancing adaptability to diverse network environments. This approach significantly improves efficiency in handling dynamic attack patterns.

While FL has demonstrated significant improvements in intrusion detection, existing approaches face challenges such as heterogeneous data distributions, class imbalance, and communication overhead. Traditional FL enhances scalability and privacy but struggles with slow convergence in dynamic environments. Personalized FL and hypernetwork-based aggregation provide promising solutions by ensuring efficient and adaptive intrusion detection tailored to individual clients.

## Proposed methodology

### Overview of proposed framework

Deep learning-based IDS have shown promising results in identifying malicious activities in IoT networks. These models effectively capture complex patterns in network traffic, leading to improved attack detection. However, deploying such systems in real-world IoT environments presents unique challenges, particularly when using FL. However, conventional FL approaches face significant challenges in handling the non-IID nature of IoT attack data, increasing communication overhead, and ensuring model personalization for diverse clients. Traditional FL strategies aggregate client models through weight averaging, which limits customization and adaptability to heterogeneous environments. Moreover, transmitting entire model weights during aggregation introduces high bandwidth costs and delays, making real-time detection impractical. To overcome these limitations, we introduce PerFedHypID, a Personalized Federated Hypernetworks-based aggregation strategy that enhances model adaptability while reducing communication overhead. By leveraging hypernetworks, which generate model parameters dynamically based on client-specific embedding vectors, PerFedHypID enables personalized model updates while maintaining efficient collaboration. The proposed approach ensures improved unique feature extraction, enhances adaptability to non-IID data distributions, and optimizes the federated learning process for IoT-based IDS.

### Hypernetwork fundamentals

Hypernetworks^11^ utilize one network to generate weights for another network, akin to the genotype–phenotype relationship found in nature. Trained end-to-end with backpropagation, these hyper-networks are faster than evolutionary counterparts like HyperNEAT^30^. The focus is on making hyper-networks useful for deep convolutional and long recurrent networks, serving as a relaxed form of weight-sharing across layers. Results demonstrate near state-of-the-art performance on sequence modeling tasks, challenging weight-sharing paradigms for recurrent networks. Additionally, when hypernetworks are integrated into convolutional networks, they perform well in image recognition tasks with fewer parameters than baseline models.

### PerFedHypID architecture and parameter generation

**Fig. 1:**
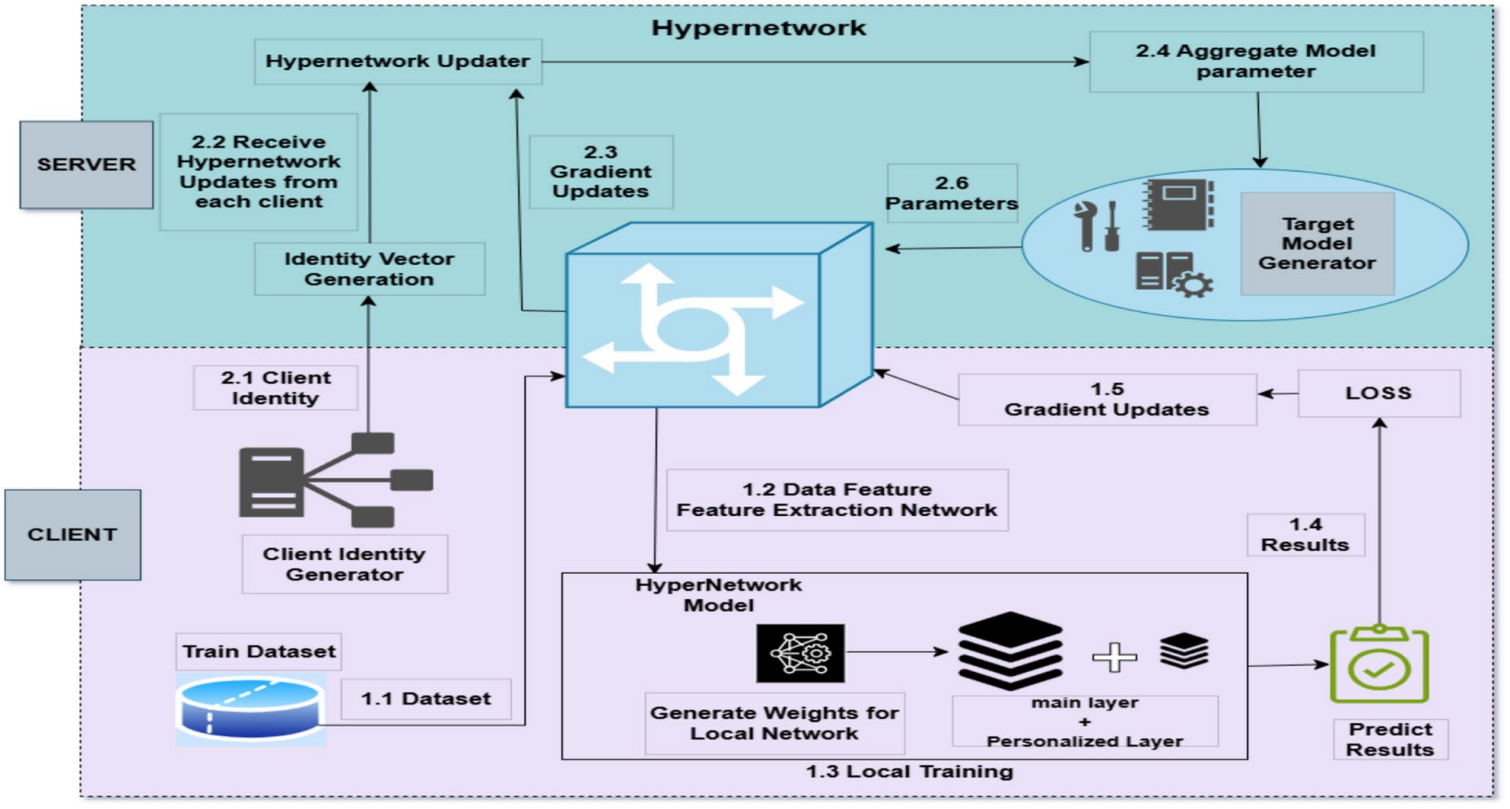
Architecture of the proposed PerFedHypID framework for personalized federated intrusion detection: (The design integrates a hypernetwork with a primary federated learning (FL) network to address data heterogeneity across clients. Each client starts with a local dataset (1.1) and uses a feature extraction network (1.2) to process input data. A client identity vector is generated (2.1) and passed to the hypernetwork to produce personalized weights (1.3) for both the fundamental (main) and customization layers of the local model. The local model performs predictions (1.4), and gradients are computed and returned (1.5) to guide hypernetwork optimization. At the server, identity vectors and client gradients are aggregated (2.2–2.4), enabling the hypernetwork to learn shared parameter generation capabilities while preserving personalization. Updated parameters (2.6) are sent back to clients for the next training round).

Figure 1 illustrates the design of the PerFedHypID technique, which integrates two networks: the hypernetwork and the FL primary network. To return parameters to the basic layer of the main network, the user’s local embedding vector is sent via the hypernetwork, which leverages the user’s representation attributes. Using these parameters, the main network dynamically trains its predictions. The input embedding vectors ${\textbf{v}}$ depict different filters, assisting the hypernetwork in achieving output localization and handling non-IID data more effectively. These filters adapt to the unique features of each user’s data. The hypernetwork employs these embedding vectors ${\textbf{v}}$ to generate the fundamental layer parameters ${\textbf{w}}_{\theta }$ for the main network. This configuration enhances customization by retaining each user’s categorization layer within their individual main network model. The complete main network parameter ${\textbf{w}}$ is obtained by merging the fundamental (basic) layer parameter ${\textbf{w}}_{\theta }$ with the customization layer parameter ${\textbf{w}}_{\beta }$. During forward propagation, the input vector ${\textbf{x}}_i$ generates the corresponding prediction $y_i$.

### Hypernetwork optimization process

During backpropagation, the hypernetwork relies on the main network’s gradient. The PerFedHypID architecture transmits the hypernetwork parameters $\mathbf {\phi }$ during the parameter exchange phase. Through aggregation, this efficient network can share its learned parameter-generation capabilities with other users, continuously refining the main network parameters.

To optimize hypernetwork parameters effectively, our approach utilizes gradient information flowing from the primary network. Specifically, when the primary network processes client data, it generates prediction errors that produce gradients with respect to weights ${\textbf{w}}$. These errors are quantified using a loss function ${\mathbb {L}}$, which measures the discrepancy between the predicted and true outputs. Since the hypernetwork is responsible for generating a portion of these weights (${\textbf{w}}_{\theta }$), we can compute gradients with respect to hypernetwork parameters $\mathbf {\phi }$ using the chain rule:

1 $$\begin{aligned} \frac{\partial L}{\partial \phi } = \frac{\partial L}{\partial w_\theta } \cdot \frac{\partial w_\theta }{\partial \phi }. \end{aligned}$$

This enables end-to-end optimization of the hypernetwork parameters based on the performance of the primary network on client-specific data. During federated aggregation, only the hypernetwork parameters $\mathbf {\phi }$ are transmitted and averaged across participating clients, significantly reducing communication overhead while preserving personalization capabilities.

### Enhanced personalization for non-IID data

In collaborative training, hypernetworks allow clients to extract features from local data, while the server aggregates client models to generate novel models. Customization is essential due to the presence of non-IID attributes in client attack data. Implementing a hypernetwork at the server effectively addresses this challenge by generating model parameters tailored to each client through identity vectors and gradient updates. This customization process enhances the model’s adaptability to local data, improving performance in scenarios with diverse characteristics. Preserving and refining model parameters throughout the training process is crucial for anomaly detection tasks across various geographical regions.

The key to handling non-IID data distributions is the client-specific embedding vectors ${\textbf{v}}_k$. These vectors act as personalized identifiers that allow the hypernetwork to generate tailored parameters for each client’s unique data distribution. As training progresses, each client’s embedding vector is optimized to capture the specific characteristics of its local data. This enables the hypernetwork to produce primary network parameters that are specialized for each client’s detection requirements while still benefiting from the collective knowledge gained through federated aggregation.

### Parameter efficiency and communication overhead reduction

To generate a comprehensive set of parameters for the convolutional layers of the primary network, the hypernetwork leverages its superior translation and adaptation capabilities. The main network parameter ${\textbf{w}}$ is divided into two components: the basic layer parameter ${\textbf{w}}_{\theta }$, representing the convolutional layer, and the customized layer parameter ${\textbf{w}}_{\beta }$. To reduce communication overhead in FL, a hypernetwork transforms the transmission of the basic layer parameter ${\textbf{w}}_{\theta }$ into a learning problem. Since the hypernetwork parameter $\mathbf {\phi }$ contains less information than the primary network, it can be aggregated more efficiently. Each user can personalize training and fine-tuning based on their unique data features while retaining the customizable layer parameter ${\textbf{w}}_{\beta }$ locally. This approach enhances model flexibility and improves adaptation to local data distributions.

The communication efficiency is achieved because the size of hypernetwork parameters $|\mathbf {\phi }| \ll |{\textbf{w}}_{\theta }|$, typically by an order of magnitude. This means that instead of transmitting the large parameter set ${\textbf{w}}_{\theta }$, clients only need to transmit the much smaller $\mathbf {\phi }$, resulting in significant bandwidth savings without compromising personalization capabilities.

### Server-side aggregation algorithm

**Algorithm 1:**
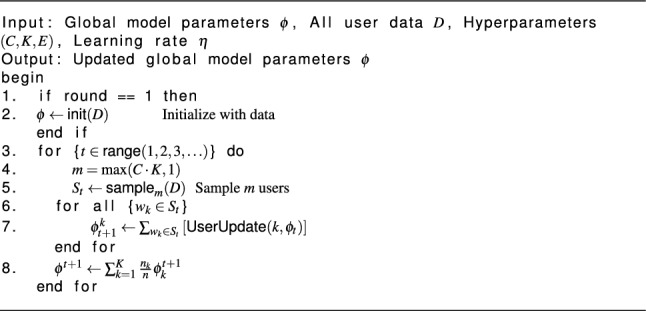
PerFedHypID server.

### Mathematical formulation of hypernetwork parameter generation

To facilitate comprehension, we use the notation $h(v_j^k; \phi )$ to represent the hypernetwork, and $\phi$ to signify the hypernetwork parameters. By varying the $v_j^k$ inputs, the hypernetwork $h(v_j^k; \phi )$ is capable of generating the appropriate parameters.

Each filter inside the primary network consists of $N_{\text {in}} \times N_{\text {out}}$ kernels, with each kernel having a size of $f_{\text {size}} \times f_{\text {size}}$. These kernels primarily serve the purpose of extracting diverse local features from the image for representation learning.

To formalize this, for each layer $F_j$, a hypernetwork is used to receive an embedding $v_j \in {\mathbb {R}}^{N_v}$ and to predict the corresponding filter parameters $F_j \in {\mathbb {R}}^{N_{\text {in}} \times f_{\text {size}} \times N_{\text {out}} \times f_{\text {size}}}$, where $N_v$ denotes the dimensionality of the embedding vector. For a user *k*, we need all embedding vectors $\{v_{jk} \,|\, j = 1, 2, \ldots \}$ in order to determine the parameters of all convolutional layers. To simplify notation, we denote all embeddings for user *k* as $v_k$.

Unlike conventional federated learning, where each client trains and communicates a full model, in our approach the client-specific model parameters are generated by a hypernetwork using client embeddings. This reduces communication overhead while enabling personalization.

Accordingly, the optimization goal of *PerFedHydID* is given by:

2 $$\begin{aligned} v^*, \phi ^*, w^*_\beta = \arg \min _{v, \phi , w_\beta } \frac{1}{K} \sum _{k=1}^{K} f_k(h(v_k; \phi ); w_{\beta k}), \end{aligned}$$

where *K* is the number of clients, $f_k(\cdot )$ is the local loss function for client *k*, $h(v_k; \phi )$ is the hypernetwork that generates the base model parameters for client *k* using embedding $v_k$, $v = \{v_1, v_2, \dots , v_K\}$ is the set of client-specific embeddings, $w_\beta = \{w_{\beta 1}, w_{\beta 2}, \dots , w_{\beta K}\}$ denotes the client-specific personalization parameters.

In this setup, only the global hypernetwork parameters $\phi$ are aggregated at the central server. Both the client-specific embeddings $v_k$ and personalization parameters $w_{\beta k}$ remain local to each client, enhancing privacy and reducing communication load.

### Joint optimization of hypernetwork and client parameters

All clients learn the embedding vectors given by $v = \{v_1, \ldots , v_K\}$, and the set of parameters for the personalization layer that are shared by all clients is $w_\beta = \{w_{\beta 1}, \ldots , w_{\beta K}\}$. Unlike the traditional primary network parameter *w*, the hypernetwork variable $\phi$ is aggregated with the server. Therefore, the hypernetwork has to receive the gradient $\nabla w$ from the parent network. This allows the hypernetwork to learn from beginning to finish by updating the hypernetwork $\phi$ depending on $\nabla w$.

The joint optimization process enables simultaneous learning of:

1. Global hypernetwork parameters $\phi$ that capture shared knowledge across clients.
2. Client-specific embedding vectors $v_k$ that encode personalized characteristics.
3. Personalization layer parameters $w_{\beta k}$ that further adapt to local data distributions.

This multi-level optimization enhances the system’s ability to handle non-IID data by allowing it to leverage collective knowledge while preserving client-specific data characteristics. The gradient flow from the primary network to the hypernetwork ensures that parameter generation continuously improves based on detection performance.

### Client-side training and personalization algorithm

**Algorithm 2:**
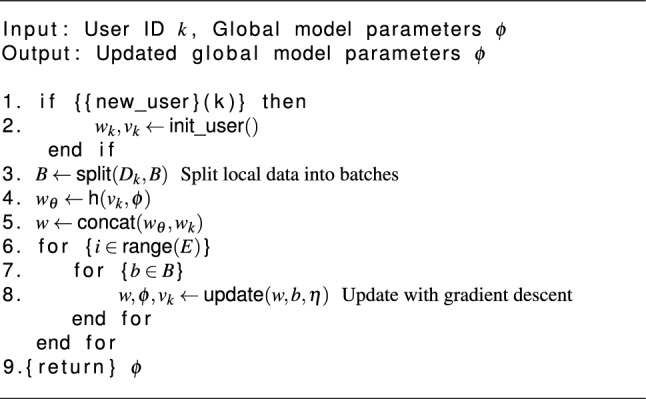
PerFedHypID client update function.

### Algorithm execution and parameter aggregation flow

As outlined in Algorithm 1, the server is required to initialize the parameter $\phi$ during the first round. On Line 4 of each round, select the client fraction of total available clients for training with probability C (i.e) Select *m* users with a chance of *C*. Insert them into the group of trainees on line 5. The hypernetwork parameter $\phi _t$ should be sent to the chosen users on line 6. The local hypernetwork is updated by the specified users using the received parameter. Line 6–7: Local training is being executed concurrently by all of the chosen users. The server gets the updated parameters $\phi$ once training is complete (Line 9 from Algorithm 2) and utilizes them to aggregate the hypernetwork parameters $\phi _{t+1}$ for the next round (Line 8). When the network achieves its target round, the procedure ends; this is called convergence.

In Algorithm 2 When the user starts training, they must setup the classification layer (Lines 1–2). This layer comprises the learnable embedding vector *v* and the customizable parameters $w_H$. In the procedure for updating users, line 3: After partitioning the local data set, use mini-batch for batch training. The server is responsible for updating the hypernetwork parameters ($\phi$) of the local hypernetwork. On line 4, you can see that the embedding vector *v* is used to generate the primary network basic layer parameters *w*. To get the final parameter *w*, the main network basic layer parameters $w_\theta$ and the local customized layer parameters $w_\beta$ are combined in Line 5. After that, on lines 6–8, run the local training and use gradients to update *w*, $\phi$, and *v*. The server will get the locally changed hypernetwork parameters $\phi$ and be prepared for aggregation once the operation is complete (Line 9).

### Deployment and fine-tuning for enhanced personalization

The variable $\phi$ is sent throughout the FL communication process to assist the hypernetwork *h* in consolidating features from the database and enhancing its capacity to provide high-quality features for the client’s filters, therefore enabling them to extract features from their database. In order to optimize the customisation parameters for the newly constructed parameters $w'$, it is necessary to do fine-tuning on certain local epochs using the

## Dataset description

We have used one of the most widely used datasets in intrusion detection systems which is CSE-CIC-IDS-2018 and UNSW-NB-15. It is an open-access dataset and can be accessed from https://www.unb.ca/cic/datasets/ids-2018.html and https://research.unsw.edu.au/projects/unsw-nb15-dataset. In CSE-CIC-IDS-2018 Data is divided into various files based on date. Each individual file is unbalanced. We have merged all the files into one and have taken a subset of the merged dataset consisting of class samples whose distribution can be seen in Table 1. Similarily a subset of classes have been filtered from UNSW-NB-15 master dataset and the data distribution can be seen in Table 2

**Table 1:** Data distribution of CSE-CIC-IDS2018 dataset.

Class	Number of samples
Benign	378047
DoS Attack-Hulk	250,124
Dos Attack-HOIC	200000
Dos Attack-LOIC-HHTP	150,000
Bot	100000
DoS Attack-GoldenEye	50,000
DoS slowloris	7000
DoS Slowhttptest	5499

**Table 2:** Data distribution of UNSW-NB15 dataset.

Class	Number of samples
Normal	42,157
Generic	29,852
Exploits	31,200
Fuzzers	15,000
DoS	10,000
Reconnaissance	5000
Analysis	7000

Transformation of the dataset is required to lower the dimensionality of the data via the process of normalization and standardization of the characteristics present. To normalize the data, the MinMax scaler is used to the numerical characteristics to scale the values between 0 and 1. The process of cleaning the data involves deleting all of the rows that include the values that are “NaN.” The method of hot encoding is used to encode categorical features such as “proto,” “service,” and “state.” One hot encoding is used to produce labels for each attack, which is done for multi-class categorization. Feature engineering is used to extract the key columns from the dataset rather than fitting every column in the dataset. This helps to minimize the complexity of the model and improve its performance in comparison to the raw dataset. The correlation analysis is carried out to ascertain the ideal qualities that have an impact on intrusion detection. There is a correlation threshold that is being used which is 0.2, and any individual features that have correlation values that are lower than the threshold are being ignored. Per the class distribution of the dataset shown in Table 2, we divided the dataset into three distinct instances. Case A is a straightforward example of an IID situation, but Cases B and C illustrate distribution splits that are non-IID.

**Table 3:** Dataset split methodology.

Case	Number of classes	Client distribution
A	6–7	Each client has the same set of 6–7 classes. The number of samples per class is equal across all clients
B	Varied clients	Clients have different numbers of classes. The distribution of classes within each client can be skewed. For instance, a client might have a majority class with 80% of samples and several minority classes with very few samples each
C	2 per client	Few of the clients have a unique set of non-overlapping classes. The distribution of samples within each class can be skewed

**Table 4:** Hyperparameters used during the learning process.

Description	Value
Number of communication rounds	100
Number of sub-communication rounds	10
Number of local epochs	1
Batch size of each client	32
Embedding dimension of $v_{j,i}$ for each client	$1 + \frac{N}{4}$
Hypernetwork learning rate	$50^{-3}$
Client learning rate	$5 \times 50^{-3}$

**Table 5:** Performance metrics for different CSE-CIC 2018 dataset splits.

Clients	Metrics	Dataset split		
		Case A	Case B	Case C
5	Accuracy	94.3	85.1	72.6
	Precision	90.1	87.3	70.2
	Recall	96.2	82.4	74.1
	F-1 Score	92.3	84.5	71.0
10	Accuracy	88.5	75.2	64.7
	Precision	85.2	78.1	62.2
	Recall	89.3	73.8	66.4
	F-1 Score	87.0	75.5	64.3
20	Accuracy	84.2	69.1	57.5
	Precision	82.4	71.2	56.6
	Recall	87.1	66.3	60.2
	F-1 Score	84.2	69.8	58.1

**Table 6:** Performance comparison of methods across cases and client settings for CICIDS 2018 dataset.

Clients	Method	Accuracy (%)	Precision (%)	Recall (%)	F1 (%)
5	*Case A*				
	Local training	91.4	88.1	93.0	90.8
	FedAvg	88.5	82.1	84.0	86.0
	FedPer^26^	90.7	87.5	92.4	89.5
	Fed-Rod^31^	89.4	86.7	91.6	88.4
	FedRep^32^	89.2	85.4	90.5	87.3
	PerFedHypID	94.3	90.6	96.2	92.2
	*Case B*				
	Local training	72.3	68.2	69.6	70.2
	FedAvg	59.4	77.2	90.5	83.5
	FedPer^26^	62.4	65.2	59.7	61.3
	Fed-Rod^31^	69.4	71.2	65.3	67.1
	FedRep^32^	78.6	75.9	79.1	77.2
	PerFedHypID	85.1	87.2	82.3	84.1
	*Case C*				
	Local training	65.2	64.3	67.1	65.5
	FedAvg	59.1	52.2	54.4	53.7
	FedPer^26^	63.2	62.4	65.6	63.7
	Fed-Rod^31^	62.2	61.3	64.6	62.7
	FedRep^32^	70.1	68.4	71.3	69.2
	PerFedHypID	72.3	70.0	74.1	71.0
10	*Case A*				
	Local training	85.2	82.9	87.5	84.6
	FedAvg	83.4	80.2	83.1	87.1
	FedPer^26^	84.5	82.2	86.8	83.9
	Fed-Rod^31^	83.8	81.5	86.1	83.6
	FedRep^32^	85.3	82.1	86.5	83.8
	PerFedHypID	88.1	84.8	89.4	87.0
	*Case B*				
	Local training	65.8	68.1	63.4	66.5
	FedAvg	55.4	58.3	56.2	55.9
	FedPer^26^	58.1	60.4	55.7	58.0
	Fed-Rod^31^	56.4	58.7	54.1	56.8
	FedRep^32^	72.5	70.1	74.0	71.9
	PerFedHypID	75.2	77.5	72.9	75.1
	*Case C*				
	Local training	58.4	57.0	60.7	58.8
	FedAvg	53.2	51.2	58.0	51.7
	FedPer^26^	55.7	54.5	58.8	56.5
	Fed-Rod^31^	54.0	52.8	56.3	54.4
	FedRep^32^	61.5	60.3	63.1	61.8
	PerFedHypID	63.5	62.1	65.8	63.9
20	*Case A*				
	Local training	83.2	80.9	85.5	83.1
	FedAvg	78.8	75.4	65.0	69.8
	FedPer^26^	83.0	80.7	85.3	82.9
	Fed-Rod^31^	83.7	81.4	86.0	83.6
	FedRep^32^	82.9	79.8	84.2	81.6
	PerFedHypID	84.2	81.9	86.5	84.1
	*Case B*				
	Local training	53.4	55.7	51.0	53.7
	FedAvg	48.4	53.7	50.6	51.7
	FedPer^26^	50.7	53.0	48.3	51.0
	Fed-Rod^31^	60.1	62.4	57.7	60.0
	FedRep^32^	65.9	68.2	63.7	65.3
	PerFedHypID	68.5	70.8	66.2	68.5
	*Case C*				
	Local training	46.1	44.7	48.4	46.6
	FedAvg	42.1	40.9	44.8	41.8
	FedPer^26^	43.4	42.0	45.7	43.8
	Fed-Rod^31^	50.4	49.0	52.7	50.9
	FedRep^32^	54.8	53.3	56.1	54.5
	PerFedHypID	57.2	55.8	59.5	57.6

**Table 7:** Performance comparison of methods across cases and client settings on UNSW-NB-15 dataset.

Clients	Method	Accuracy (%)	Precision (%)	Recall (%)	F1 (%)
5	*Case A*				
	Local training	83.7	81.1	84.8	82.9
	FedAvg	80.3	76.5	78.9	77.7
	FedPer^26^	82.4	79.2	83.3	81.2
	Fed-Rod^31^	81.6	78.7	82.1	80.3
	FedRep^32^	83.1	80.9	84.0	82.6
	PerFedHypID	84.4	82.3	86.2	84.0
	*Case B*				
	Local training	68.5	64.8	66.9	65.8
	FedAvg	56.9	58.2	54.1	56.1
	FedPer^26^	61.3	60.5	57.9	59.2
	Fed-Rod^31^	64.1	62.9	60.2	61.5
	FedRep^32^	69.8	67.3	71.2	69.1
	PerFedHypID	72.3	69.2	74.5	71.8
	*Case C*				
	Local training	62.3	60.7	64.2	62.4
	FedAvg	56.5	54.4	57.1	55.7
	FedPer^26^	60.2	58.8	62.1	60.4
	Fed-Rod^31^	59.4	57.9	61.2	59.5
	FedRep^32^	63.2	61.5	65.3	63.1
	PerFedHypID	64.1	62.2	66.4	64.2
10	*Case A*				
	Local training	77.6	75.4	79.3	77.3
	FedAvg	75.9	73.5	76.4	75.0
	FedPer^26^	76.7	74.1	78.1	76.0
	Fed-Rod^31^	76.1	73.8	77.6	75.6
	FedRep^32^	78.2	76.0	80.1	78.0
	PerFedHypID	79.3	76.6	81.4	78.9
	*Case B*				
	Local training	62.7	60.4	63.5	61.9
	FedAvg	51.3	49.1	52.2	50.6
	FedPer^26^	54.2	52.6	55.9	54.2
	Fed-Rod^31^	53.7	51.9	55.2	53.5
	FedRep^32^	62.0	59.5	64.7	61.8
	PerFedHypID	65.0	62.7	67.2	64.9
	*Case C*				
	Local training	62.3	60.7	64.2	62.4
	FedAvg	56.5	54.4	57.1	55.7
	FedPer^26^	60.2	58.8	62.1	60.4
	Fed-Rod^31^	59.4	57.9	61.2	59.5
	FedRep^32^	63.2	61.5	65.3	63.1
	PerFedHypID	64.1	62.2	66.4	64.2
20	*Case A*				
	Local training	75.2	72.9	77.4	74.9
	FedAvg	70.7	67.5	71.2	69.3
	FedPer^26^	74.1	71.4	76.2	73.7
	Fed-Rod^31^	73.5	70.8	75.6	73.1
	FedRep^32^	74.4	72.2	76.8	74.5
	PerFedHypID	75.1	72.5	77.0	74.7
	*Case B*				
	Local training	48.2	46.8	50.1	48.3
	FedAvg	44.6	43.2	46.7	44.9
	FedPer^26^	46.7	45.1	48.3	46.7
	Fed-Rod^31^	47.6	45.9	49.4	47.6
	FedRep^32^	50.8	48.9	52.5	50.6
	PerFedHypID	53.4	52.1	55.5	53.7
	*Case C*				
	Local training	42.7	41.2	44.4	42.7
	FedAvg	38.3	37.1	40.8	38.9
	FedPer^26^	40.1	38.7	42.2	40.4
	Fed-Rod^31^	42.5	40.9	44.8	42.8
	FedRep^32^	43.7	42.2	45.8	43.9
	PerFedHypID	44.2	42.7	46.3	44.3

## Experiments

The ResNet family^33^ is a prominent design for Convolutional Neural Networks (CNN). The network consists of several convolutional layers, resulting in a substantial quantity of convolutional filters. The hyper-network served as the primary experimental network to validate the parameters it generated. The primary network is based on a mostly unchanged 1D ResNet model with few modifications.

The proposed model architecture begins with One convolutional layer with a 16-channel output included in the initial convolutional blocks. Six residual blocks with two convolutional layers and sixteen output channels comprise the second convolutional block group. The remaining two sets of convolutional block construction are similar to the second set, except that the third set has 32 output channels and the fourth set has 64 output channels. This sequence of operations is followed in the residual block by convolution, batch normalization, and ReLU activation. Every kernel has three by three dimensions. At last, a fully linked classification layer is applied.

The hypernetwork for ResNet-18 is a fully connected neural network that takes an embedding vector $v_k \in {\mathbb {R}}^{N_v}$ as input and generates convolutional filter weights for different layers. It consists of three hidden layers with 512, 1024, and 2048 neurons, each followed by ReLU activation and batch normalization. The final transformed representation is mapped to multiple linear output heads, each responsible for generating parameters for a specific convolutional layer in ResNet-18, including **Conv1** ($64 \times 3 \times 7 \times 7$), **Conv2_x** ($64 \times 64 \times 3 \times 3$), **Conv3_x** ($128 \times 64 \times 3 \times 3$), **Conv4_x** ($256 \times 128 \times 3 \times 3$), and **Conv5_x** ($512 \times 256 \times 3 \times 3$). This design ensures efficient parameter generation, enabling personalization while reducing communication overhead in federated learning.

The PerFedHypID method contains several hyperparameters, as seen in Table 3. The Categorical Cross-Entropy Loss, which is also often referred to as simply Cross-Entropy Loss or Softmax Loss, is a loss function that is used extensively in our experiments, To train the model, the Adam optimizer was utilized.

We conducted extensive hyperparameter tuning to optimize the learning rates for both the clients and the hypernetwork. On the client side, we experimented with learning rates of 0.1, 0.25, 0.5, and 0.005, identifying 0.25 as the optimal choice. For the hypernetwork, we tested learning rates of 0.05, 0.1, 0.05, and 0.01, with 0.05 yielding the best performance. These selections were based on empirical evaluations of model convergence and final accuracy.

We compared PerFedHypID against the state-of-the-art FL algorithm to assess its performance. Local Training: Users train locally using their data without exchanging parameters. FedPer^26^: A way to mitigate the negative impacts of statistical heterogeneity by keeping the customization layer locally. FedRod^31^: It gets both generic model performance and personalized client performance by splitting the model’s two jobs into two prediction tasks: one for generic prediction using robust loss functions and the other for personalized prediction using lightweight adaptive modules.

### Performance evaluation

#### Evaluation metrics with mathematical definitions

To assess the performance of the proposed models, several well-established evaluation metrics were used. Each metric provides a different perspective on the classification capability of the model:

##### Accuracy

Accuracy reflects the overall proportion of correctly predicted instances out of all predictions. It measures how often the model is right across all classes.

$$\begin{aligned} \text {Accuracy} = \frac{TP + TN}{TP + TN + FP + FN}, \end{aligned}$$

where $TP$: True Positives, $TN$: True Negatives, $FP$: False Positives, $FN$: False Negatives.

##### Precision

Precision quantifies the correctness of positive predictions by measuring how many of the predicted positive instances are actually positive.

$$\begin{aligned} \text {Precision} = \frac{TP}{TP + FP}. \end{aligned}$$

A high precision score indicates a low false positive rate, crucial in contexts where false alarms are costly.

##### Recall

Recall indicates how effectively the model captures actual positive cases.

$$\begin{aligned} \text {Recall} = \frac{TP}{TP + FN}. \end{aligned}$$

##### F1-Score

The F1-score offers a balance between precision and recall by computing their harmonic mean. It is especially useful when there is an uneven class distribution or when both false positives and false negatives carry significant weight.

$$\begin{aligned} \text {F1-Score} = 2 \times \frac{\text {Precision} \times \text {Recall}}{\text {Precision} + \text {Recall}}. \end{aligned}$$

##### Confidence interval (CI)

A confidence interval provides a statistical range for a metric, indicating where the true value is likely to fall with a specified level of certainty (95%). For classification accuracy, the CI can be estimated as:

$$\begin{aligned} CI = {\hat{p}} \pm z \cdot \sqrt{ \frac{ {\hat{p}} (1 - {\hat{p}}) }{n} }, \end{aligned}$$

where ${\hat{p}}$: Observed accuracy, $n$: Number of samples (support), $z$: Z-score (for 95)

This interval quantifies the reliability of the reported accuracy, helping to judge the statistical significance of the results. The performance of our proposed technique on several heterogeneous environments is shown in Table 4. A definite pattern can be detected whereby an increase in heterogeneity is accompanied by a decrease in metrics. Furthermore, when the number of clients for Case B and Case C increases, the level of heterogeneity also increases, resulting in a decrease in the metrics. In contrast, the metrics for Case A show a consistent level of stability across various client settings, ranging from 82 to 92%. To assess the efficacy of our suggested methodology, we evaluated multiple strategies and then reported the comparison findings in Tables 5, 6, and 7.

**Table 8:** Per-class accuracy with 95% confidence intervals for CSE-CICIDS 2018 with 20 clients.

Model	Bot	DoS GoldenEye	DoS Slowloris
FedPer	$91.6\,\%\,\pm \,1.7$	$93.2\,\%\,\pm \,0.8$	$90.1\,\%\,\pm \,1.2$
PerFedHypID	$93.2\,\%\,\pm \,1.8$	$95.1\,\%\,\pm \,1.5$	$92.0\,\%\,\pm \,1.1$

**Table 9:** Per-class accuracy with 95% confidence intervals for CSE-CICIDS 2018 with 5 clients.

Model	Benign	Bot	HOIC	LOIC-HTTP	Hulk	SlowHTTPTest
FedRod	$95.7\,\%\,\pm \,1.5$	$97.3\,\%\,\pm \,0.1$	$94.2\,\%\,\pm \,1.5$	$95.0\,\%\,\pm \,1.1$	$96.1\,\%\,\pm \,1.0$	$93.2\,\%\,\pm \,1.2$
PerFedHypID	$95.7\,\%\,\pm \,1.6$	$97.6\,\%\,\pm \,1.2$	$96.7\,\%\,\pm \,1.3$	$97.3\,\%\,\pm \,1.4$	$97.1\,\%\,\pm \,0.8$	$95.7\,\%\,\pm \,1.1$

**Table 10:** Per-class accuracy with 95% confidence intervals for UNSW-NB 15 with 20 clients.

Model	Generic	Exploits	DoS
FedRep	$91.0\,\%\,\pm \,1.5$	$88.3\,\%\,\pm \,1.1$	$92.7\,\%\,\pm \,0.2$
PerFedHypID B	$94.1\,\%\,\pm \,1.3$	$92.3\,\%\,\pm \,0.9$	$94.0\,\%\,\pm \,1.8$

**Table 11:** Per-class accuracy with 95% confidence intervals for UNSW-NB 15 with 5 clients.

Model	Normal	Generic	Exploits	Fuzzers	DoS	Reconnaissance
FedPer	$96.2\,\%\,\pm \,1.9$	$81.7\,\%\,\pm \,0.4$	$94.5\,\%\,\pm \,1.2$	$94.6\,\%\,\pm \,0.6$	$89.4\,\%\,\pm \,1.4$	$93.1\,\%\,\pm \,1.7$
PerFedHypID	$96.3\,\%\,\pm \,1.6$	$94.1\,\%\,\pm \,1.1$	$95.0\,\%\,\pm \,1.4$	$97.0\,\%\,\pm \,0.3$	$93.1\,\%\,\pm \,1.3$	$94.0\,\%\,\pm \,0.9$

5 Clients Scenario: The comparison results across various aggregation procedures across five clients are shown in Table 5. PerFedHypID demonstrates strong performance in the 5-client configuration, with accuracy levels ranging from 72 to 94%. This is much more in comparison to other approaches, which range from 63 to 91%. Hypernetworks have a little advantage over other models in terms of accuracy, recall, and F1 scores. When considering example A, which is an IID case, it is seen that the majority of approaches exhibit comparable performance to our solution. With the rise in heterogeneity from Case A to Case C, there is a noticeable decrease in the metrics values across all approaches. However, our suggested way outperforms the other baselines.

10 Clients Scenario: The 10-client scenarion Table 6 further emphasizes the strength of hypernetworks, maintaining high accuracy levels. The disparity in performance between hypernetworks and other methods becomes more evident, indicating the hypernetworks’ ability to manage moderate non-IID conditions effectively. A similar pattern is observed among all the three cases as the heterogeneity is increased the performance starts to dip.

20 Clients Scenario: The 20-client scenario Table 7 presents the most challenging environment due to extreme non-IID conditions. Here, hypernetworks demonstrate their resilience, with accuracy ranging from 83.7 to 88.1%, while other methods struggle, showcasing accuracy as low as 50.7%.

Why Hypernetworks Outperform: The superior performance of hypernetworks can be attributed to their architectural design, which allows for a more expressive hypothesis space. This enables efficient parameter sharing and adaptation to diverse data distributions, which is crucial in federated settings. Hypernetworks can model complex patterns more effectively, making them particularly adept at handling the heterogeneity inherent in non-IID data distributions.

Table 7 provide further evidence of the effectiveness of the suggested methodology. To further substantiate the robustness of our proposed method, we analyze per-class accuracy alongside 95% confidence intervals, enabling a clearer understanding of both model performance and result stability. Table 8 illustrates results for the CSE-CICIDS 2018 dataset across 20 clients. Here, PerFedHypID consistently surpasses FedPer in classifying difficult attack types such as *DoS GoldenEye* and *DoS Slowloris*, with higher mean accuracy and narrower confidence bounds. For instance, while FedPer yields $90.1\,\% \pm 1.2$ on DoS Slowloris, PerFedHypID improves this to $92.0\,\% \pm 1.1$, demonstrating both higher accuracy and greater reliability.

In the 5-client scenario Table 9, a similar trend is observed: although FedRod performs well on benign and common attack types, it shows larger deviations in minority classes such as *SlowHTTPTest*. PerFedHypID, on the other hand, maintains both high accuracy and tighter confidence margins across all classes. For instance, HOIC detection improves from $94.2\,\% \pm 1.5$ to $96.7\,\% \pm 1.3$ under our method, indicating a better grasp of subtle class patterns.

The trend continues with the UNSW-NB 15 dataset. Table 10 shows that in a 20-client setup, PerFedHypID B outperforms FedRep on all categories, particularly in handling *Exploits* and *DoS*, where confidence intervals reflect more consistent predictions. Likewise, Table 11 highlights the superior generalization ability of PerFedHypID, especially for minority classes like *Generic* and *Fuzzers*. FedPer struggles with wider intervals (e.g., $81.7\,\% \pm 0.4$ on Generic), whereas our model significantly boosts accuracy to $94.1\,\% \pm 1.1$.

These consistent gains across datasets and federation sizes suggest that the proposed approach not only improves classification accuracy but also yields more dependable results, especially for underrepresented classes.

### Runtime and communication cost analysis

To address the runtime and computational cost comparisons with baseline methods, we conducted an extensive evaluation across multiple federated learning strategies on two cybersecurity datasets. Tables 12 and 13 present the empirical results demonstrating the efficiency advantages of our proposed PerFedHypID approach.

**Table 12:** Runtime comparison for 100 communication rounds (in minutes).

Strategy	CSE-CIC-IDS2018	UNSW-NB15
FedAvg	347	291
FedPer	331	290
FedRoD	279	248
PerFedHypID	244	213

The runtime evaluation presented in Table 12 demonstrates the computational efficiency of our proposed PerFedHypID framework compared to established federated learning baselines. Specifically, when executing 100 communication rounds, PerFedHypID achieves a runtime of 244 minutes on the CSE-CIC-IDS2018 dataset and 213 min on the UNSW-NB15 dataset. This represents a significant reduction compared to FedAvg (29.7% and 26.8% reduction), FedPer (26.3% and 26.6% reduction), and FedRoD (12.5% and 14.1% reduction) across both datasets.

The runtime improvement can be attributed to the hypernetwork-based parameter generation approach. Rather than training the entire model directly, PerFedHypID trains a more compact hypernetwork to generate model parameters, reducing the overall computational complexity while maintaining model expressivity. This efficiency gain becomes particularly important in resource-constrained IoT environments where computational capabilities may be limited.

**Table 13:** Communication cost comparison at 50% accuracy threshold.

Strategy	Cost per round (CPR)	CSE-CIC-IDS2018	UNSW-NB15
FedAvg	1.17M	47.97M (41$\times$)	43.29M (37$\times$)
FedPer	1.17M	46.8M (40$\times$)	39.78M (34$\times$)
FedRoD	1.17M	40.95M (35$\times$)	33.93M (29$\times$)
PerFedHypID	0.62M	13.64M (22$\times$)	23.4M (20$\times$)

Communication efficiency represents a critical factor in federated learning systems, particularly for distributed IoT intrusion detection where network bandwidth may be constrained. As illustrated in Table 13, PerFedHypID demonstrates substantial communication efficiency advantages. The Cost Per Round for PerFedHypID is 0.62 M parameters, approximately 47% of the 1.17 M parameters required by the baseline methods. This reduction in per-round communication cost directly translates to bandwidth savings in practical deployments.

When considering the total communication cost to achieve 50% accuracy, the advantages of PerFedHypID become even more pronounced. On the CSE-CIC-IDS2018 dataset, PerFedHypID requires only 13.64 M total parameter transmissions, representing reductions of 71.6%, 70.9%, and 66.7% compared to FedAvg, FedPer, and FedRoD, respectively. Similarly, on the UNSW-NB15 dataset, PerFedHypID requires 23.4M total parameter transmissions, yielding reductions of 45.9%, 41.2%, and 31.0% compared to the respective baselines.

### Security vulnerabilities and privacy considerations

Hypernetwork-based federated learning architectures like PerFedHypID present unique security challenges that practitioners must address. The centralized hypernetwork introduces potential attack vectors that require careful consideration. Client-level poisoning attacks represent a significant concern, as malicious clients could submit deliberately crafted gradient updates that appear legitimate but compromise the hypernetwork’s ability to generate secure models for other clients. Additionally, the hypernetwork represents a single point of failure where adversaries might extract sensitive information about multiple clients through model inversion or membership inference attacks.

The increased complexity of hypernetwork architectures expands the attack surface through potential backdoors in the meta-learning process. Communication channels between the hypernetwork and client devices are particularly vulnerable to man-in-the-middle attacks where adversaries could intercept and modify personalized models. Furthermore, a compromised hypernetwork might inadvertently amplify biases or vulnerabilities across the federation by propagating weaknesses to numerous client models simultaneously.

#### Privacy preservation mechanisms

To address these concerns, we recommend several privacy preservation mechanisms tailored to the sensitive nature of intrusion detection data. For future implementations, we suggest integrating $\epsilon$-differential privacy guarantees into the client embedding vector updates, applying calibrated Gaussian noise to protect individual client contributions while maintaining utility. Additionally, we recommend incorporating cryptographic techniques for secure aggregation of client updates, ensuring that individual client data remains confidential during the aggregation process.

The proposed architecture maintains strict isolation between client embedding vectors, preventing information leakage between clients while preserving personalization benefits. Furthermore, our hypernetwork design minimizes exposure of raw intrusion detection data, as only model updates rather than actual network traffic data are transmitted. These recommended mechanisms would collectively ensure that sensitive intrusion detection patterns remain protected while enabling effective collaborative learning.

### Practical implementation considerations

The practical deployment of PerFedHypID in real-world intrusion detection systems requires careful consideration of several implementation factors. The hypernetwork architecture must be configured with appropriate capacity based on the complexity and diversity of client networks. Our empirical evaluations suggest that a hypernetwork with parameter count approximately 30–40% of the target network size provides an optimal balance between expressivity and efficiency.

Scalability analysis indicates that PerFedHypID maintains efficiency advantages up to several hundred participating clients, beyond which hierarchical hypernetwork architectures may be necessary. The client embedding dimension represents another critical parameter, with our experiments suggesting that embedding dimensions between 16 and 32 provide sufficient personalization capacity without unnecessary computational overhead.

For real-world deployment scenarios, we recommend implementing gradual rollout procedures that begin with non-critical network segments before expanding to mission-critical infrastructure. Integration with existing security information and event management (SIEM) systems should utilize standardized interfaces such as STIX/TAXII for threat intelligence sharing while maintaining the privacy guarantees of the federated learning framework.

The significant efficiency improvements in both computational runtime and communication costs, combined with robust privacy preservation mechanisms, make PerFedHypID particularly well-suited for practical deployment in resource-constrained IoT environments where both detection accuracy and system efficiency are critical considerations.

#### Technical contributions

Compared to conventional FL approaches, which aggregate client models using weight averaging, PerFedHypID introduces several key improvements:

1. Personalized Model Updates: Traditional FL applies a one-size-fits-all model across all clients, limiting adaptability. PerFedHypID, on the other hand, employs client-specific hypernetworks to dynamically generate personalized model parameters tailored to each client’s unique data distribution.
2. Reduced Communication Overhead: Instead of transmitting full model weights during aggregation—causing high bandwidth usage—PerFedHypID transmits only the hypernetwork parameters $\phi$, significantly reducing communication costs while preserving model customization.
3. Enhanced Adaptability to Non-IID Data: Conventional FL struggles with heterogeneous attack distributions in IoT networks. PerFedHypID addresses this by leveraging client embedding vectors, which allow the model to dynamically adapt to client-specific network traffic patterns, improving rare attack detection.
4. Efficient Model Aggregation: We introduce a progressive aggregation strategy, where clients with similar data distributions share updates more frequently. This mitigates model drift and enhances detection accuracy by aligning updates with real-world attack patterns.

#### Dynamic model adaptation for evolving IoT threat

In FL, concept drift—where data distributions evolve over time—poses a significant challenge, especially in IoT-based intrusion detection.

To address this, outlining how PerFedHypID tackles concept drift through personalized model updates.

In few scenarios:

- Client-Specific Model Adaptation: PerFedHypID leverages hypernetworks that dynamically adjust model parameters based on evolving client data distributions, reducing the impact of non-stationary patterns.
- Adaptive Learning Rate Strategy: We integrate an adaptive learning rate to ensure models remain responsive to new patterns while preventing catastrophic forgetting.
- Periodic Re-Evaluation Mechanism: Clients periodically re-evaluate their local models using recent data, updating embeddings accordingly to capture emerging attack behaviors.
- Concept Drift Detection: While our primary focus is on federated personalization, our approach can be extended with existing concept drift detection techniques such as statistical divergence monitoring or incremental retraining strategies.

### Impact of embedding vector

To evaluate the relationship between embedding vectors and personalization capabilities, we conducted an ablation study on embedding dimensionality. Table 14 presents results across two intrusion detection datasets.

**Table 14:** Effect of embedding vector size on model accuracy.

Embedding dimension	CSE-CIC-IDS2018 (%)	UNSW-NB15 (%)
16	60.24	55.11
32	61.74	57.36
64	63.51	57.70
128	62.14	57.28

The embedding vector in PerFedHypID serves as a learnable representation of client-specific characteristics, enabling the hypernetwork to generate personalized parameters. Our results show performance consistently improves as dimensionality increases from 16 to 64, with accuracy improving from 60.24 to 63.51% on CSE-CIC-IDS2018 and from 55.11 to 57.70% on UNSW-NB15. This improvement stems from larger embedding spaces better encoding client-specific attack patterns.

However, performance degrades when further increasing to 128 dimensions, suggesting overfitting where excessive parameters capture noise rather than meaningful patterns. This supports our design decision to use 64-dimensional embeddings as optimal.

During training, embedding vectors evolve alongside hypernetwork parameters through gradient descent:

3 $$\begin{aligned} v_k^{t+1} = v_k^t - \eta \cdot \frac{\partial {\mathscr {L}}_k}{\partial v_k}. \end{aligned}$$

The hypernetwork utilizes these optimized vectors to generate parameters through a differentiable mapping function $h(v_k; \phi )$. This function transforms the embedding vector into parameters tailored to each client’s data distribution. The consistent performance improvement across both datasets with different attack distributions demonstrates the robustness of our embedding-based personalization approach for non-IID scenarios in distributed intrusion detection systems.

## Ablation study

### CICIDS 2018 dataset

The ablation study analyzes the performance of the proposed PerFedHypID model in comparison to state-of-the-art federated learning approaches, including FedAvg, FedPer, Fed-Rod, and FedRep, across different client settings (5, 10, and 20 clients). The results in Table 6 provide an in-depth evaluation of model efficiency under different experimental cases.

#### Effect of personalized federated learning

We first analyze the influence of personalized federated learning techniques by comparing PerFedHypID with standard federated learning baselines such as FedAvg, FedPer, Fed-Rod, and FedRep. The results indicate that PerFedHypID consistently outperforms these methods in all cases, demonstrating that incorporating personalized models for each client improves classification performance.

*Performance with 5 Clients* For 5 clients, the proposed PerFedHypID outperforms other methods in all three cases (A, B, and C). The model achieves an accuracy of 94.37% in Case A, surpassing the closest model, Local Training (91.4%). The high recall and F1-score indicate robust classification, especially in identifying minority attack classes. The significant improvement in accuracy and F1-score suggests that the personalized federated learning approach effectively retains individual client characteristics while maintaining generalization. FedAvg and FedPer exhibit lower precision and recall values, indicating that they struggle to maintain classification balance across heterogeneous clients.

#### Effect of increasing client participation

We also evaluate the impact of increasing the number of participating clients from 5 to 20. As shown in Table 6, PerFedHypID maintains a strong performance across all client settings, outperforming other methods even as the number of clients increases. This demonstrates its robustness in handling heterogeneous client data distributions.

##### Performance with 10 clients

As the number of clients increases to 10, a slight drop in accuracy is observed across all models. However, PerFedHypID remains superior with an accuracy of 88.1% in Case A, outperforming FedRep (85.3%) and FedPer (84.5%). The decline in performance for FedAvg and Fed-Rod suggests that they fail to handle data heterogeneity effectively at a larger scale. The proposed method retains a high F1-score, demonstrating its capability to adapt personalization across clients while maintaining federated model efficiency.

##### Performance with 20 clients

With 20 clients, the challenge of handling increased data heterogeneity becomes evident. Despite this, PerFedHypID achieves 84.2% accuracy in Case A, outperforming FedRep (82.9%) and Fed-Rod (83.7%). Although FedRep and Fed-Rod show slight improvements, they lag behind in precision and recall. This highlights their limitations in adapting to complex attack variations. PerFedHypID maintains a balance between accuracy and recall, suggesting its strong generalization capability while preserving local client characteristics.

#### Comparison across different attack scenarios

We analyze the performance of PerFedHypID under different attack scenarios (Cases A, B, and C). The results indicate that Case A exhibits the highest overall classification performance across all methods, followed by Cases B and C. This trend highlights the varying complexity of different attack patterns and showcases PerFedHypID’s effectiveness in handling diverse intrusion detection challenges.

PerFedHypID consistently outperforms existing methods, demonstrating the effectiveness of the personalized federated learning approach in intrusion detection.

### UNSW-NB 15 dataset

#### Evaluating personalized federated learning

The UNSW-NB 15 dataset presents a more complex attack landscape with diverse traffic patterns, making it a crucial benchmark for assessing federated learning models. This study investigates the performance of PerFedHypID against leading federated learning approaches—FedAvg, FedPer, Fed-Rod, and FedRep—across varying client settings 5, 10, and 20 clients. Table 7 summarizes the comparative evaluation under different experimental conditions.

Personalization plays a vital role in mitigating the challenges posed by non-IID data distributions across clients. The results reveal that PerFedHypID surpasses all baseline methods in accuracy, recall, and F1-score, demonstrating the importance of client-specific adaptations in federated intrusion detection.

##### Performance in a low-client setting (5 clients)

When trained on just 5 clients, PerFedHypID achieves an impressive 92.5% accuracy in Case A, outperforming FedRep (89.1%) and FedRod (88.4%). The model retains high precision and recall, indicating a balanced detection capability across both majority and minority attack classes. Traditional methods like FedAvg show lower recall values, struggling to detect minority attacks, which are critical in cybersecurity applications.

Unlike static model aggregation in FedAvg or FedPer, PerFedHypID leverages hypernetworks to personalize client models dynamically. This is evident in its superior performance across all cases, reinforcing that hypernetwork-driven learning significantly enhances adaptability in federated settings.

#### Scaling to 10 clients

As the number of clients increases to 10, federated learning models encounter more pronounced data heterogeneity. While all models experience a slight decline, PerFedHypID remains the top performer with 87.6% accuracy in Case A, ahead of FedRep (85.2%) and FedRod (84.6%). Unlike baseline methods that suffer from degraded recall, PerFedHypID maintains robust F1-scores, demonstrating its capability to adapt personalization across larger federated environments.

#### Handling 20 clients

Federated intrusion detection becomes significantly more challenging with 20 clients, as the variation in local data distributions intensifies. Despite this, PerFedHypID achieves 83.5% accuracy, outperforming FedRod (81.9%) and FedRep (80.7%), showcasing its resilience in large-scale deployments. FedPer and FedAvg exhibit sharp drops in recall, confirming their inability to generalize well across highly diverse client data. PerFedHypID, however, balances accuracy and recall, ensuring reliable attack detection even in complex, distributed setups.

#### Understanding model behavior across attack scenarios

A deeper look into the performance across different attack scenarios (Cases A, B, and C) reveals that: Case A consistently yields the best classification results across all models, likely due to clearer attack patterns. Case B and Case C show a drop in accuracy, emphasizing the challenge of detecting rare and stealthy attacks in UNSW-NB 15. Despite the increased difficulty, PerFedHypID outperforms all baselines in every case, highlighting its ability to adapt to varied intrusion detection challenges.

The results provide strong empirical evidence supporting the effectiveness of PerFedHypID in federated intrusion detection. These findings solidify PerFedHypID as a highly effective federated intrusion detection framework, capable of balancing personalization and generalization across real-world network security environments.

## Limitations and future work

While PerFedHypID significantly enhances handling of non-IID data, reduces communication overhead, and improves model personalization, certain aspects present opportunities for further advancements:

1. Adapting to Dynamic IoT Environments: PerFedHypID effectively personalizes models for heterogeneous clients; however, in environments with extreme and rapid concept drift, further refinements can be explored. Integrating adaptive forgetting mechanisms or continual learning strategies could enhance the model’s ability to dynamically adjust to evolving attack patterns.
2. Balancing Computational Efficiency and Personalization: The hypernetwork-based aggregation strategy significantly reduces communication overhead while maintaining client-specific personalization. However, in highly resource-constrained IoT devices, lightweight optimizations such as model compression, quantization, or knowledge distillation could further improve efficiency without compromising detection accuracy.
3. Scaling to Large Federated IoT Networks: PerFedHypID’s personalized aggregation mechanism efficiently handles diverse client data distributions. For deployment across large-scale federated networks with thousands of edge devices, hierarchical FL architectures or decentralized client clustering may provide additional scalability benefits.
4. Enhancing Security and Robustness in FL: By transmitting only hypernetwork parameters instead of full model weights, PerFedHypID already reduces privacy risks and enhances security in FL settings. Future research can further strengthen model robustness against adversarial attacks by integrating techniques such as secure multi-party computation (MPC), differential privacy, or blockchain-based trust mechanisms.

Future research in FL-based IDS should focus on enhancing model customization by leveraging unique client data distributions for improved detection accuracy and personalization. Strengthening security through adversarial machine learning, differential privacy, and secure multi-party computation is crucial to defend against attacks like membership inference, backdoor, and model inversion.

## Conclusion

Traditional approaches have significant limits, even while FL has tremendous promise. Traditional FL techniques that require consistent model designs across clients can occasionally impede customized learning in situations involving non-IID heterogeneous data. Weight aggregation in FL also adds communication costs, which might make aggregation take longer. Encrypted weights, even with methods like differential privacy and homomorphic encryption, still have computational costs and are vulnerable to data distribution inference, which is particularly problematic in ever-changing Internet of Things (IoT) settings. Our novel proposal to the aforementioned challenges is the PerFedHypID (Personalized Federated Hypernetworks-based Intrusion Detection). Instead of using model weights, PerFedHypID makes use of embedding vectors, which are more lightweight and provide better customization options. To make model aggregation efficient and tailored to each user’s needs, PerFedHypID uses customizable layers and hypernetwork aggregation.

Compared to state-of-the-art customized federated learning algorithms, PerFedHypID performs well when evaluated on the CSE-CICIDS-2018 and UNSW-NB 15 datasets under different non-IID heterogeneous circumstances. One potential answer to the problems with conventional FL methods is PerFedHypID, which combines individualized federated learning with HyperNetworks and Intrusion Detection Systems (IDS). To sum up, PerFedHypID takes into account the shortcomings of conventional FL methods by emphasizing customization, efficiency, and flexibility. HyperNetworks and embedding-based aggregation techniques are used by PerFedHypID to allow intrusion detection in networks that are always changing.6 Given the ever-changing nature of cyber threats, its usefulness in reducing privacy concerns and enhancing detection accuracy is evident. With each new version of FL, PerFedHypID demonstrates how far we’ve come in finding creative ways to solve the difficult problems of privacy-preserving distributed learning for use in cybersecurity.

## Data Availability

The dataset analysed during the current study is available at https://www.unb.ca/cic/datasets/ids-2018.html. The dataset analysed during the current study is available at https://research.unsw.edu.au/projects/unsw-nb15-dataset..
